# An aegerolysin-like protein from Heliothis virescens ascovirus 3h (HvAV-3h) shows immune suppression and antibacterial activity

**DOI:** 10.1099/jgv.0.002107

**Published:** 2025-05-29

**Authors:** Heba A. H. Zaghloul, Zhengkun Xiao, Jun Tang, Ting Xiao, Jiajun Gao, Jianjun Hu, Guo-Hua Huang

**Affiliations:** 1 Hunan Provincial Key Laboratory for Biology and Control of Plant Diseases and Insect Pests, College of Plant Protection, Hunan Agricultural University, Changsha, Hunan 410128, PR China; 2 Department of Botany and Microbiology, Faculty of Science, Alexandria University, Alexandria 21511, Egypt; 3 Yuelushan Laboratory, Hunan Agricultural University, Changsha, Hunan 410128, PR China

**Keywords:** aegerolysin, antibacterial, ascovirus, HvAV-3h, immune suppression, pore formation

## Abstract

Aegerolysins are lipid-binding proteins associated with multiple functions, including membrane pore-formation, insecticidal toxicity and defence against predators. Whilst distributed over the kingdoms of the Tree of Life, ascoviruses are the only representative viruses that encode an aegerolysin-like protein. Ascoviruses are entomopathogenic and possess a large dsDNA genome. The present study aimed to functionally characterize the aegerolysin-like protein of Heliothis virescens ascovirus 3h (HvAV-3h), encoded by ORF85, and to explore its potential roles in the interaction between the ascovirus and its host. Our results demonstrate the importance of this species-specific protein to HvAV-3h replication in host cells. *In vivo*, silencing of this gene for 12–72 h significantly increased the expression of some innate immunity-associated genes, including Toll (114-fold), IMD (44.7-fold) and Hopscotch (22.9-fold). In parallel, we detected significant gradual increases in MyD88 and Relish and decreases in PIAS. Moreover, histopathological analyses of infected larval tissues indicated reduced tissue damage after 72 h of ORF85 gene silencing. The prokaryotic expression of the HvAV-3h aegerolysin, followed by feeding to third-instar *Spodoptera exigua* larvae for 24 or 48 h led to significant reductions in larval weight. Moreover, the *in vitro* treatment demonstrated a bactericidal action against *Lysinibacillus xylanilyticus*, a bacterial resident of some insect guts. Overall, our findings suggest that the protein encoded by ORF85 is associated with the pathogenicity of HvAV-3h and its ability to replicate in host cells. Additionally, aegerolysin may inhibit or kill specific bacterial species in the host microbiome during infection, potentially modulating the host immune response.

## Introduction

The aegerolysin family members are characterized by their beta-structured proteins, small molecular weights (15–20 kDa), small isoelectric points and stability over a wide pH range [1,2]. The Asp-haemolysin represents the first aegerolysin-like protein isolated and sequenced [3,4]. This protein was found to be associated with *Aspergillus fumigatus* pathogenesis [5]. Subsequently, many other similar proteins were discovered and assigned to aegerolysins [1]. Nowadays, aegerolysin proteins are present in diverse kingdoms in the Tree of Life. These kingdoms include Fungi, Bacteria, Plantae and Animalia [1,6]. However, ascoviruses uniquely encode aegerolysin-like proteins in their genomes [1,7, 8]. For example, two aegerolysins, ORF85 and ORF029, are encoded by ascovirus Heliothis virescens ascovirus 3h (HvAV-3h) and Trichoplusia ni ascovirus (TnAV-6a1), respectively [9]. However, the role of these proteins in ascovirus pathogenesis and potential biotechnological applications has not been explored before. Therefore, the current study aims to characterize a putative aegerolysin-like gene (ORF85) encoded by the HvAV-3h genome during the *in vivo* infection of lepidopteran larvae.

Ascoviruses are insect-killing pathogens that target lepidopteran insects in agricultural fields. They possess a large and circular dsDNA genome [10]. The cytopathological changes induced in the ascovirus-infected insect are very characteristic. For example, within 2–3 days of infection, the infected insect haemolymph (blood) turns milky white, easily recognized by the naked eye. This colour develops due to the enormous accumulation of sac-like vesicles known as ‘virion-containing vesicles’ in the infected haemolymph. These vesicles form after nucleus destruction and cell hypertrophy [10,11]. Each hypertrophied cell divides into about 20–30 vesicles. Interestingly, these vesicles occupied by ascovirus virions keep circulating for weeks in the insect’s open circulatory system. During this long time period, the chances of virus transmission increase as these viruses transmit mainly mechanically and little by feeding. Mechanical transmission occurs when a female wasp becomes a carrier to the vesicles or virions after contaminating its ovipositor during the deposition of eggs or probing stage of some infected insect hosts [11,12]. The discovery that the expression of a virus-encoded caspase enzyme caused nuclear lysis and led to vesicle formation may be the most intriguing aspect of ascovirus replication. This finding highlights the paradoxical ability of ascoviruses to modulate the apoptosis pathway in cell death to form these vesicles for replication and transmission [9,13, 14].

Recent transcriptomic studies conducted on some ascoviruses like Spodoptera frugiperda ascovirus 1a (SfAV-1a), TnAV-6a1 and HvAV-3h during *in vivo* infection of their lepidopteran hosts revealed the high expression levels of aegerolysin-like genes in HvAV and TnAV [9,15, 16]. Moreover, an extended TnAV-6a1 transcriptomic study for 3 weeks revealed the continuation of these high expression levels after this long time of infection. On the other hand, the SfAV-1a genome was missing a homologous gene [9,17]. Therefore, aegerolysin-like proteins are species-specific rather than core proteins in ascoviruses [18]. Ascovirus species differences are generally more likely caused by species-specific proteins [14].

Tissue tropism is amongst the main differences between SfAV and other ascovirus species. For example, SfAV was found to invade mainly the insect’s fat body tissue. On the other hand, TnAV and HvAV possessed a wider tissue tropism, as they were able to invade several tissues, including the fat body, epidermis and tracheal matrix. Furthermore, the SfAV transmission appears to be limited only to species in the genus *Spodoptera*, whilst HvAV and TnAV were able to infect species belonging to several genera [11]. Whether aegerolysin proteins play a role in this tissue tropism and host preference differences is not defined yet.

Previous studies on some aegerolysins derived from fungi revealed the multifunctional nature of these proteins. For example, erylysin A, pleurotolysin A2 and ostreolysin A6 derived from *Pleurotus* mushrooms were found to bind and permeabilize lipid membranes. On the other hand, they possessed insecticidal toxicity against the larval stage of western corn rootworm and Colorado potato beetle. Moreover, they enhanced the fruiting stage initiation in *Pleurotus ostreatus* [19]. Therefore, to determine the potential role(s) of this viral aegerolysin-like protein during an ascovirus infection in living insects and other potential biotechnological applications, we explored the possible association of this protein in diverse biological processes like virus replication, immunity evasion, insect toxicity, antimicrobial action and cell lysis. The main findings of this study support the importance of the HvAV-3h aegerolysin-like protein in HvAV-3h replication. Moreover, it confirms the multifunctional nature of this small-size protein (9.67 kDa) during ascovirus *in vivo* infection of lepidopteran larvae and highlights a biotechnologically novel and underexplored source for this class of proteins.

## Methods

### *In silico* analysis of ORF85 protein properties and phylogenetic analysis

The amino acid sequence of HvAV-3h ORF85 was analysed using ProtParam available through https://web.expasy.org/protparam/ and the I-TASSER server [20,21] available through https://zhanggroup.org/. The analyses included the protein’s estimated molecular weight, theoretical pI, amino acid composition, instability index and protein 3D structure. Furthermore, the protein structural model was used to predict the protein function based on similarity with other proteins in the Protein Data Bank (PDB). Finally, blastp was used to identify homologous proteins in the NCBI database, followed by phylogenetic analysis using the neighbour-joining (NJ) method combined with 1,000 bootstrapping for phylogenetic tree construction using mega6 software. Protein multiple sequence alignment with structurally similar proteins was made by clustalw. The alignment was visualized using ESPript 3.0 [22] at https://espript.ibcp.fr/ESPript/cgi-bin/ESPript.cgi. On the other hand, the aegerolysin protein was screened for antimicrobial peptide (AMP) activity and cell-penetrating motifs using CAMPR4 (Collection of Anti-Microbial Peptides) [23] accessed on the 15th of August 2024 through https://camp.bicnirrh.res.in/ and CellPPD [24,25], respectively.

### Virus and insects

HvAV-3h-infected haemolymph was used for all infections in this study following the procedure described by Yu *et al*. [15]. Briefly, the early/late third-instar larvae of *Spodoptera exigua* and *Helicoverpa armigera* were injected with HvAV-3h using a small-size pin contaminated with infected haemolymph to mimic the wasp mechanical transmission [14]. The infected larvae were fed on an artificial diet and maintained at 27 ℃±1 ℃. The insects were grown for 16 h in light and 8 h in dark.

### The synthesis of ORF85 dsRNA and gene silencing

The ORF85 and EGFP genes were amplified using 2×Hieff Ultra-Rapid HotStart PCR master mix (with dye) and the PCR programme: 95 °C for 3 min and 35 cycles of 95 °C, 55 °C and 72 °C for 15, 15 and 30 s, respectively. The final extension step was at 72 °C for 5 min. The primer pair (ORF85dsF and ORF85dsR) used in the amplification reaction was designed using Primer blast (NCBI) and was modified by adding the T7 polymerase promoter sequence at the 5′-end (Table 1). The dsRNA was synthesized using the T7 RiboMAX™ Express RNAi System (Promega, Madison, WI, USA) following the manufacturer’s instructions. To silence the ORF85 gene, the third-instar larvae of *S. exigua* were injected with HvAV-3h virions as described above, and the infection was followed for three successive days. The prepared dsRNA was diluted (0.5 µg µl^−1^), and each infected larva was injected with 2 µl. The control larvae were injected with EGFP-dsRNA. The silenced larva’s RNA was collected after 12, 24, 48 and 72 h post-silencing (hps). The total RNA was collected at the described time points (12–72 hps) from three biological replicates. Each biological replicate consisted of three silenced *S. exigua* larvae. The EGFP-silenced larvae were treated similarly. The total RNA was extracted following the instructions described by AG RNAex Pro Reagent. Finally, to confirm the gene silencing step, the mRNA level of ORF85 was measured in samples using quantitative reverse transcription PCR (qRT-PCR) and designed primers (ORF85qpcr-F and ORF85qpcr-R) described in Table 1. The qRT-PCR was conducted in a 10 µl reaction volume following the instructions of Hieff™ qPCR SYBR^®^ Green Master Mix (YEASEN, China).

**Table 1.** Primers designed or used in this study

Primer name	Primer sequence (5′–3′)
**For cloning**	
ORF85-F	ATGAAGTTCTCGGAATCGAATAAG
ORF85-R	TTAAGGACTTATAATTTTATGACACAC
**For expression**	
ExORF85F	GGATCCATGAAGTTCTCGGAATCGAATAAG
ExORF85R	AAGCTTAGGACTTATAATTTTATGACACAC
ExEGFPF	GCGGCCGCATGGTGAGCAAGGGCGAGGA
ExEGFPR	CTCGAGCTTGTACAGCTCGTCCATGC
**For silencing**	
ORF85dsF	TAATACGACTCACTATAGGGCAACCGTGTCGTTTTCCGTG
ORF85dsR	TAATACGACTCACTATAGGGGTCGTAGTACAACGGACCGC
EGFPdsFEGFPdsR	TAATACGACTCACTATAGGGGCTGACCCTGAAGTTCATCTGTAATACGACTCACTATAGGGGAACTCCAGCAGGACCATGT
**For qPCR**	
ORF85qpcr-F	CGGAATCGAATAAGGCCGTG
ORF85qpcr-R	CGCCAACTCGTTGCCATTTT
Se-Toll1-F	ATTGTCGGAACTGCAGTCGT
Se-Toll1-R	TCCGAGTCTTCAGGCAGAGA
Se-Toll2-F	GACACACACCAGAGGCGTAA
Se-Toll2-R	TTTGGCAATCGCATGCTACG
Se-Toll3-F	ATTTTATTGGCGACGCGAGC
Se-Toll3-R	GGAAACTTGGGTCGGTGGAT
Se-Toll4-F	GTGTGTCCGGTGAGAATGGT
Se-Toll4-R	ACTCGTTGTTCATGGAGCGT
Se-IMD-F	TGCCAGTCGTTATTCGAGGC
Se-IMD-R	GACAAAGAACACGAGGCAGC
Se-Hop-F	GCCAGTGAAGAGAGGTGTCC
Se-Hop-R	ACTCGCCAACTCCACGAAAT
Se-JH-F	GTTTGGGTATGCCACAAGTG
Se-JH-R	CAACTAATGTCCTTACCGTGG
Se-EcR-F	ACGCGCTGAACAGAACAGG
Se-EcR-R	ACTGTGAGGATCGTCATCTC
Relish-F	GCAAGTGCATTGTACTCTGC
Relish-R	GCGCGTAATTGTTGTTTGTGG
MyD88-F	CGCGCTGAGTGTATTCTGC
MyD88-R	GTTCACCTGTGGTAGTGTTG
PIAS-F	GTCTCCTAGAAACTTCGTGTG
PIAS-R	GGTGATAGTTTCACTAGGGAGG
3h-MCP-F	AATCAAATCCGTACGCCTGAAGG
3h-MCP-R	TTCGCACCAATGTCAACGGAACG

### Virus replication

The effect on virus DNA replication level was estimated using qRT-PCR as described by Li *et al.* [26]. The DNA was isolated from three biological replicates of HvAV-3h-infected larvae or HvAV-3h-infected and ORF85-silenced larvae after 12, 24, 48 and 72 hps.

### Histopathological changes in ORF85-silenced larvae

We examined the histopathological changes developed after ORF85 gene silencing in third-instar *H. armigera* larvae infected with HvAV-3h. Briefly, 12 h HvAV-3h-infected *H. armigera* larvae were injected with one microgram of ORF85-specific or EGFP-specific dsRNA (described above). The dsRNA-injected larvae were fixed after 12, 24, 48 and 72 hps. The collected larvae were transverse-sectioned, haematoxylin-eosin stained and examined using an inverted microscope. Healthy (not injected), mock-injected (healthy haemolymph was used for injection), HvAV-3h-infected and dsEGFP-injected larvae collected at the same time points served as controls. The histopathological sample processing and examination were made as described by Yu *et al.* [15]. Three biological replicates were examined in each case.

### Quantification of *S. exigua* innate immunity-associated genes

The effect of aegerolysin-like ORF85 gene silencing on the expression level of some innate immunity-associated genes identified in the *S. exigua* genome [27,28] was tested using qRT-PCR. Briefly, representative immunity genes that function as receptors or effectors in the Toll, IMD and JAK-STAT pathways were selected for this analysis. The qRT-PCR reaction solutions were prepared following the manufacturer’s instructions of the ChamQ Universal SYBR qPCR Master Mix (Vazyme Biotech Co., Ltd., Nanjing, China). The examined genes were Toll1 (MW286450.1), Toll2 (MW286451.1), Toll3 (MW286452.1), Toll4 (MW286453.1), IMD-like (JQ710732.1), Hop (CAH0695933.1), MyD88 (MG837005.1), Relish (HQ680461.1) and a PIAS-like gene identified in the *S. exigua* transcriptome.

### Cloning of HvAV-3h ORF85, an aegerolysin-like protein

Two pairs of primers were designed based on the nucleotide sequence of the aegerolysin-like gene (protein AYD68205) available through the HvAV-3h genome (NCBI accession number KU170628) to facilitate sequencing and cloning of the gene of interest (Table 1). First, ORF85 was PCR-amplified from cDNA following the instructions of 2×Rapid Taq Master Mix (Vazyme Biotech Co., Ltd., Nanjing, China). The PCR cycling conditions were as follows: initial denaturation at 95 °C for 3 min, followed by 35 cycles of 95 °C for 15 s, 60 °C for 15 s and 72 °C for 15 s.

The PCR product was then gel purified and ligated into the pGEM-T easy vector [Promega (Beijing) Biotech Co., Ltd.], following the manufacturer’s instructions. The plasmids isolated from three white clones were sequenced through TsingKe Biological Technology Co., Ltd. (Changsha, China). Validated clones were cultured to extract the verified plasmids using a SteadyPure plasmid DNA extraction kit (Accurate Biology), followed by plasmid digestion using two restriction enzymes, BamHI and HindIII [Promega (Beijing) Biotech Co.]. The obtained ORF85 was ligated in BamHI- and HindIII-digested pET-28a (Novagen, Inc., Darmstadt, Hessen, Germany) using T4 DNA ligase. The constructed expression vector was denoted pET-28a-ORF85.

### Protein expression, purification and validation

The expression vector pET-28a-ORF85 was transformed into *Escherichia coli* BL21 (DE3) strain by heat shock at 42 °C. Liquid Luria–Bertani (LB) culture medium (supplemented with kanamycin 50 µg ml^−1^) of transformed bacterial cells was initially grown in a small volume, and the next day, 3 ml was used to inoculate 150 ml of the same culture medium for protein production. For protein expression induction, 1 mM of isopropyl-*β*-d-thiogalactoside (IPTG) (Sigma, St. Louis, MO, USA) was added for 18 h at 37 °C and 250 r.p.m. incubation conditions. The bacterial cells were collected by centrifugation (5 min, 6,000 ***g***, 4 °C). Afterwards, the collected cell pellet was suspended in 22.5 ml lysis buffer (50 mM NaH_2_PO_4_×2H_2_O, 300 mM NaCl, 10 mM imidazole) supplemented with lysozyme and 225 µl PMSF. The suspended bacterial cells were disrupted by sonication (pulsed, 5 s on, 9 s off, 18 min). The homogenate was further centrifuged (20 min, 8000 ***g***, 4 °C). The His-tagged aegerolysin was detected as a soluble protein in the supernatant fraction and isolated by following the instructions of Ni-NTA Sefinose (TM) Resin (Settled Resin) (Sangon Biotech, Shanghai). Non-specifically bound proteins were washed away with wash buffer (50 mM NaH_2_PO_4_×2H_2_O, 300 mM NaCl, 20 mM imidazole, pH=8). The His-tagged aegerolysin protein was eluted in elution buffer (10 mM, 50 mM, 100 mM, 200 mM, 300 mM imidazole, pH=8). Protein samples were checked on the surface of 4–20% SDS-PAGE (MeilunBio, China). Protein concentration was determined using a plate reader at 562 nm (Tecan SPARK, Austria) using an Aidlab Pierce^TM^ BCA protein assay kit (Thermo Scientific^™^, Shanghai, China). The protein was concentrated using Amicon^®^ Ultra, 3 kDa MWCO (Merck). Finally, Western blotting using His-tag antibodies (Abmart) was made to validate the protein band following the methods described by [29]. A synthetic EGFP-encoding gene was cloned by following the same methods and using the same cloning vector (described above) for subsequent protein expression and purification using the same described approach and reagents. The His-tag-fused EGFP-purified protein served as a control for any residual chemicals that remained during the purification process.

### Examinations of prokaryotic expressed HvAV-3h aegerolysin-like protein putative biological functions

We conducted a series of experiments to examine the possible biological activity/roles associated with this protein during the HvAV-3h course of infection. The same amounts and concentrations of EGFP-purified protein and PBS buffer only served as negative controls for the following experiments.

### Haemolytic activity

The haemolytic activity of the agerolysin-like protein was determined on the surface of 5% goat blood agar using the well-diffusion method. Briefly, 90 µl of the purified aegerolysin protein (~100 µg ml^−1^) dissolved in PBS buffer was added to the well on the blood agar, followed by 30 min incubation at 4 °C to allow protein or buffer diffusion, followed by 24 h incubation at 25 °C. The appearance of a clear zone or no zone formation surrounding the well was reported as positive and negative haemolytic activity, respectively [30]. The experiment was conducted in triplicates.

### Insecticidal activity and gut bacterial load

To determine the feeding insecticidal toxicity of the purified HvAV-3h aegerolysin protein, we fed the early third-instar larvae of *S. exigua* for 24 or 48 h on *Vicia faba* leaf discs dipped in purified aegerolysin (100 µg ml^−1^) dissolved in PBS. Three biological replicates were included in each case (15 larvae per replicate). The mortality percentage, larval weight and *S. exigua* juvenile hormone epoxide hydrolase (*JHEH*) (GenBank: DQ431847.1) and ecdysone receptor (*EcR*) (GenBank: EU426551.1) relative gene expression level were recorded after a duration of 24 or 48 h of feeding following the manufacturer’s instructions of ChamQ Universal SYBR qPCR Master Mix (Vazyme Biotech Co., Ltd., Nanjing, China). On the other hand, the total bacterial load was quantified in mid-guts collected from aegerolysin-fed larvae. For the quantification of *JHEH* and *EcR* relative expression levels as well as bacterial load, we incorporated three biological replicates, with each replicate consisting of three larvae. The 16S rRNA universal primers (338 F:5′-ACTCCTACGGGAGGCAGCAG-3′ and 806 R:5′-GGACTACHVGGGTWTCTAAT-3′) were used for gene amplification following the methods described in [31,32].

### *In vitro* antibacterial activity

The antibacterial effect of the purified protein was tested on the surface of nutrient agar or LB agar plates using the well-diffusion method (described above). About 0.1 ml from overnight bacterial culture was used to inoculate the plate, followed by a well-cut and addition of 50–90 µl of the purified protein (~100–340 µg ml^−1^). The inoculated plates were incubated at 37 °C for 24 h. The experiment was repeated three times for each bacterial strain. Filter paper saturated with chloramphenicol (25 mg ml^−1^) or ampicillin (10 µg/disc) served as positive controls.

### Data analysis

The unpaired Student *t*-test was used to analyse the difference between the obtained means. A value of *P<0.05 refers to the degree of statistical significance. The data was analysed by GraphPad Prism 10 (GraphPad Software, San Diego, CA, USA). For relative gene expression, the glyceraldehyde 3-phosphate dehydrogenase (GAPDH) gene was used for normalization in each sample. The 2−ΔΔCT method was used for relative expression level calculations [33]. The one-way ANOVA followed by the Tukey test was used to compare the obtained means of larval weight, bacterial load and hormonal gene expression level. The test was conducted using the SPSS 22.0 statistical software.

## Results

### The structural and phylogenetic characteristics of the HvAV-3h ORF85 protein indicate its similarity to aegerolysin toxins found in both prokaryotic and eukaryotic organisms

**Fig. 1.**
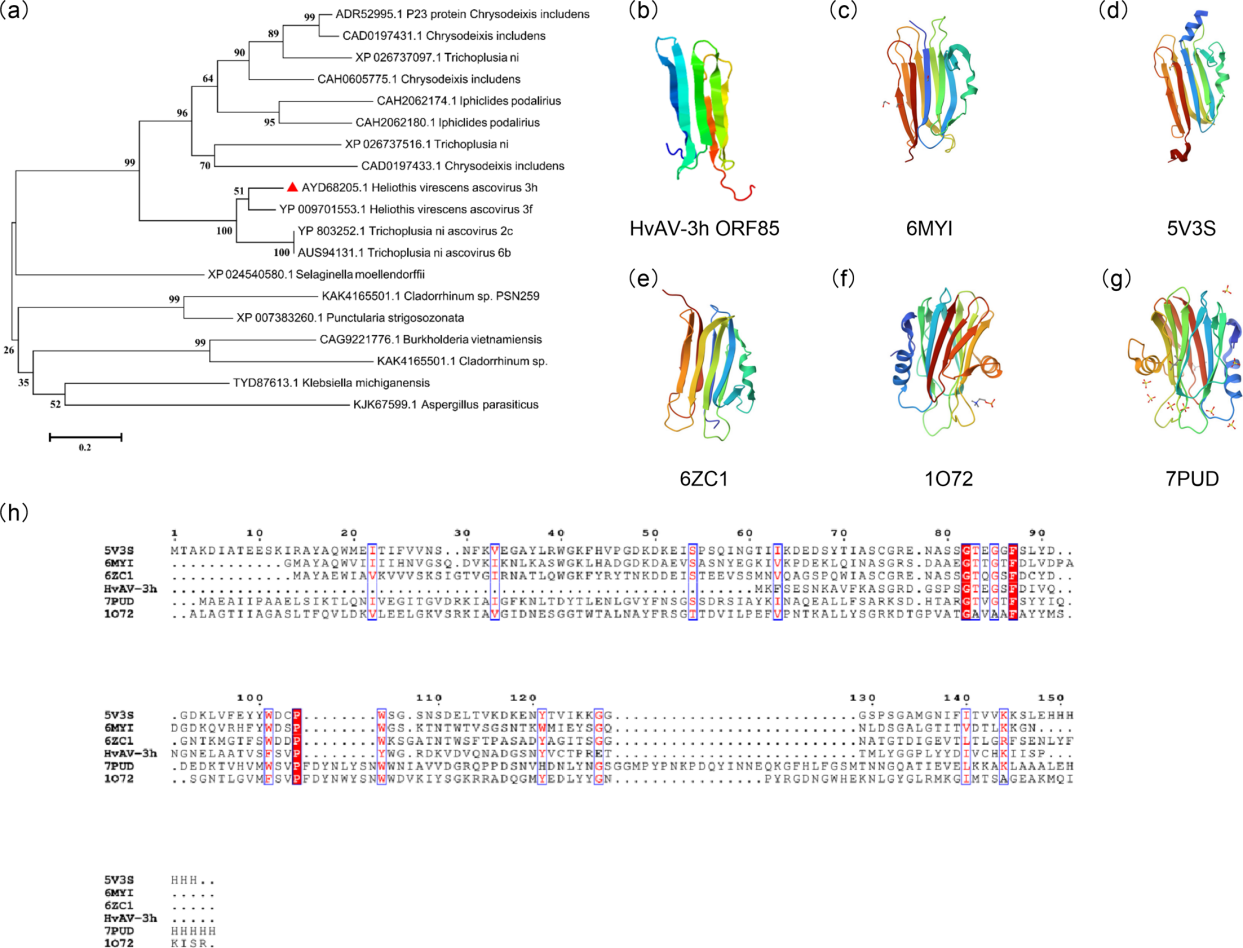
*In silico* analyses of HvAV-3h aegerolysin-like protein encoded by ORF85. (**a**) Phylogenetic analysis of HvAV-3h aegerolysin protein using the NJ method combined with 1,000 bootstrapping. The tree was constructed using mega6 software. The red triangle refers to the HvAV-3h aegerolysin-like protein (NCBI protein ID number AYD68205.1). (**b**) The tertiary structure (3D) of HvAV-3h ORF85 predicted by I-TASSER at https://zhanggroup.org/. The presented model C-score=0.24. (**c–g**) The top five proteins that have a similar structure to HvAV-3h aegerolysin protein in the PDB were *P. ostreatus* OstreolysinA (PDB number 6MYI), *A. faecalis* IP-1A insecticidal toxin (PDB number 5V3S), RahU protein from *P. aeruginosa* (PDB number 6ZC1), a pore-forming protein StnII from *S. helianthus* (PDB number 1O72) and a pore-forming protein bryoporin from the moss *P. patens* (PDB number 7PUD). (**h**) Multiple sequence alignment of the HvAV-3h aegerolysin protein with the top five structurally similar proteins. The alignment was visualized using ESPript 3.0 [22] at https://espript.ibcp.fr/ESPript/cgi-bin/ESPript.cgi.

Based on the HvAV-3h ORF85 amino acid sequence, the estimated molecular weight of the protein encoded by this gene is 9.67 kDa, with a theoretical pI=5.15. The protein has a serine- (11.2%) and glycine (10.1%)-rich sequence and an instability index value of 33.22, which identifies the protein as stable. On the other hand, the protein tertiary structure (3D) (Fig. 1) revealed that the predicted structural model is close to other protein structures in the PDB database. The top five proteins that have a similar structure were *P. ostreatus* OstreolysinA (PDB number 6MYI), *Alcaligenes faecalis* IP-1A insecticidal toxin (PDB number 5V3S), RahU protein from *Pseudomonas aeruginosa* (PDB number 6ZC1), a pore-forming protein sticholysin II (StnII) from *Stichodactyla helianthus* (PDB number 1O72) and a pore-forming protein bryoporin from the moss *Physcomitrium patens* (PDB number 7PUD). In addition, screening the HvAV-3h aegerolysin-like protein sequence using CAMPR4 revealed that this protein has a 0.65 probability of possessing an AMP activity. Moreover, the assessment of the viral aegerolysin-like protein for its ability to penetrate cells, conducted using CellPPD, revealed a cell-penetrating protein (CPP) amino acid motif characterized by the sequence LALLA. However, we did not detect a putative homologue for cell-penetrating peptides in the CellPPD database that match the HvAV-3h aegerolysin peptide sequences. Finally, the phylogenetic analysis using the protein sequence of HvAV-3h ORF85 revealed that only HvAV and TnAV species encode homologous aegerolysin-like proteins (Fig. 1). Moreover, the viral aegerolysin protein was homologous to proteins identified in some lepidopteran pests, namely, *Chrysodeixis includens*, *Iphiclides podalirius* and *Trichoplusia ni*. This result implies that the evolutionary source for HvAV-3h aegerolysin might be its lepidopteran insect hosts.

### Suppression of HvAV-3h ORF85 negatively affected viral replication and triggered the immune response of the host

**Fig. 2.**
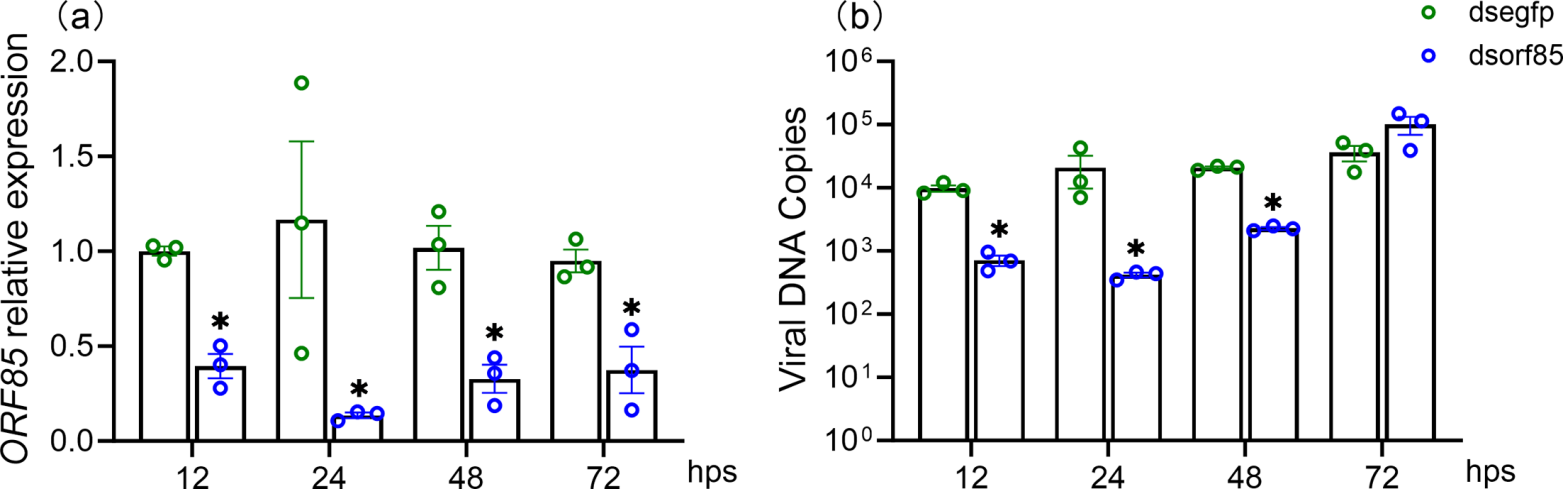
qRT-PCR quantification of the HvAV-3h DNA copies in infected *S. exigua* third-instar larvae after silencing of the virus ORF85 gene expression. (**a**) Validation of HvAV-3h ORF85 relative expression level decrease in HvAV-3h-infected *S. exigua* larvae after 12, 24, 48 or 72 hps. (**b**) Quantification of virus DNA copies after 12, 24, 48 and 72 hps. Larvae injected with dsEGFP served as a control. Three biological replicates were included at each time point. Error bars represent the standard error values. The unpaired Student *t*-test was used to analyse the significant difference between the obtained means. The star (*) symbol refers to *P*<0.05.

To examine the function of HvAV-3h ORF85, identified as an aegerolysin-like protein, for HvAV-3h viral replication, we silenced this gene via RNA interference using gene-specific dsRNA. The HvAV-3h infection, followed by ORF85 gene silencing for different times (12, 24, 48 and 72 h), revealed the importance of this gene for virus replication. First, the injection of HvAV-3h-infected * S. exigua* larvae with dsRNA significantly silenced ORF85 transcripts as reflected in all the tested time points (Fig. 2a), in comparison to HvAV-3h-infected and dsEGFP-injected larvae after 12–72 hps. This result reflects the success of this technique in HvAV-3h ORF85 gene silencing *in vivo* for third-instar HvAV-3h-infected *S. exigua* larvae. Second, the viral DNA load in dsORF85-injected larvae demonstrated a significant decrease in virus replication induced after this gene silencing from 12 to 72 hps. At 72 hps, we detected a statistically non-significant increase in the viral DNA load (Fig. 2b). This result implies that silencing of ORF85 reduces HvAV-3h replication and infection levels.

**Fig. 3.**
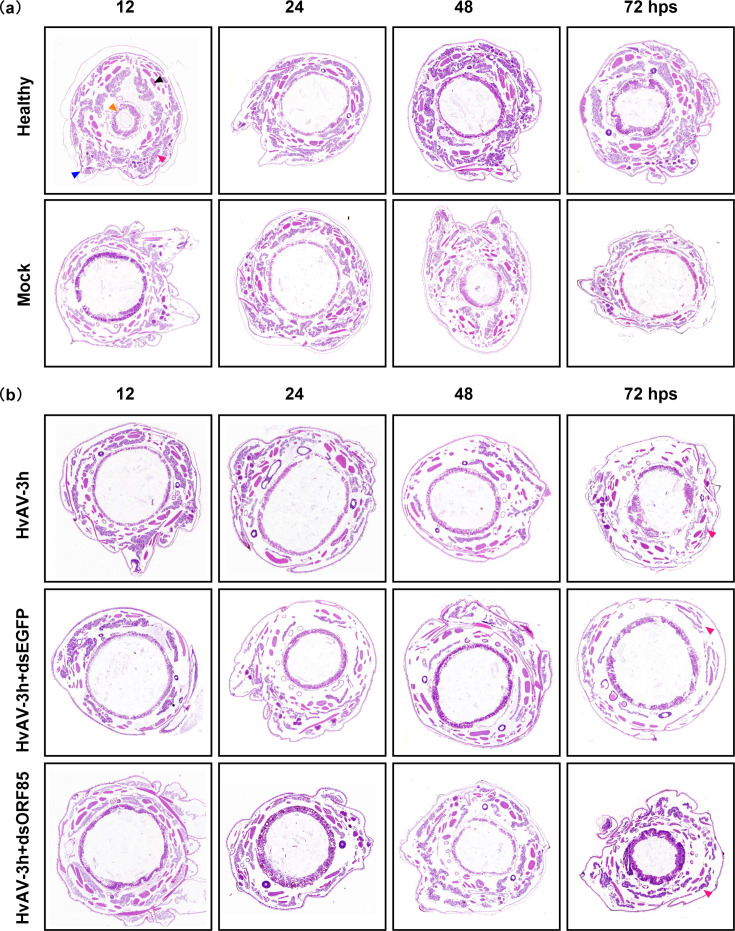
Histopathological changes induced in *H. armigera* third-instar larvae after HvAV-3h infection and ORF85 silencing using gene-specific dsRNA. The a and b panels demonstrate the insect sections obtained after 12, 24, 48 and 72 hps. The transverse sections were stained with haematoxylin–eosin stain. These histopathological changes were induced after infection with HvAV-3h only or HvAV-3h for 12 h, followed by injection of HvAV-3h dsORF85 or dsEGFP. The pink triangle refers to the fat body, the blue triangle refers to the cuticle, the black triangle refers to muscles and the brown triangle refers to the midgut. The healthy panel refers to larvae that did not receive any haemolymph injection, whereas the mock refers to larvae that were injected with healthy haemolymph. Compared to the HvAV-3h and HvAV-3h+dsEGFP panels at the same time point, the HvAV-3h+dsORF85 panel at 72 hps showed that aegerolysin gene silencing resulted in less damage of the fat body cells (pink triangle).

The decline in viral replication associated with silencing the ORF85 was also evident in the observation of diminished damage in the tissues of the infected host (Fig. 3a). We noticed during our histopathological examinations of infected host tissues after ORF85 silencing that the host tissue status was comparable to the healthy or mock control larvae from 12 to 72 hps (Fig. 3a). The healthy and mock *H. armigera* transverse sections revealed the normal tissue appearance characterized by light-stained fat bodies and a dark-stained midgut circle in the middle of the transverse section. On the contrary, following the histopathological alterations after HvAV-3h infection for the same period (12–72 hpi) demonstrated a pathological change in fat bodies that become loose with infection progress (Fig. 3b). This result reflects a decrease in the virus’s ability to cause more damage to the host tissues after silencing of ORF85.

**Fig. 4.**
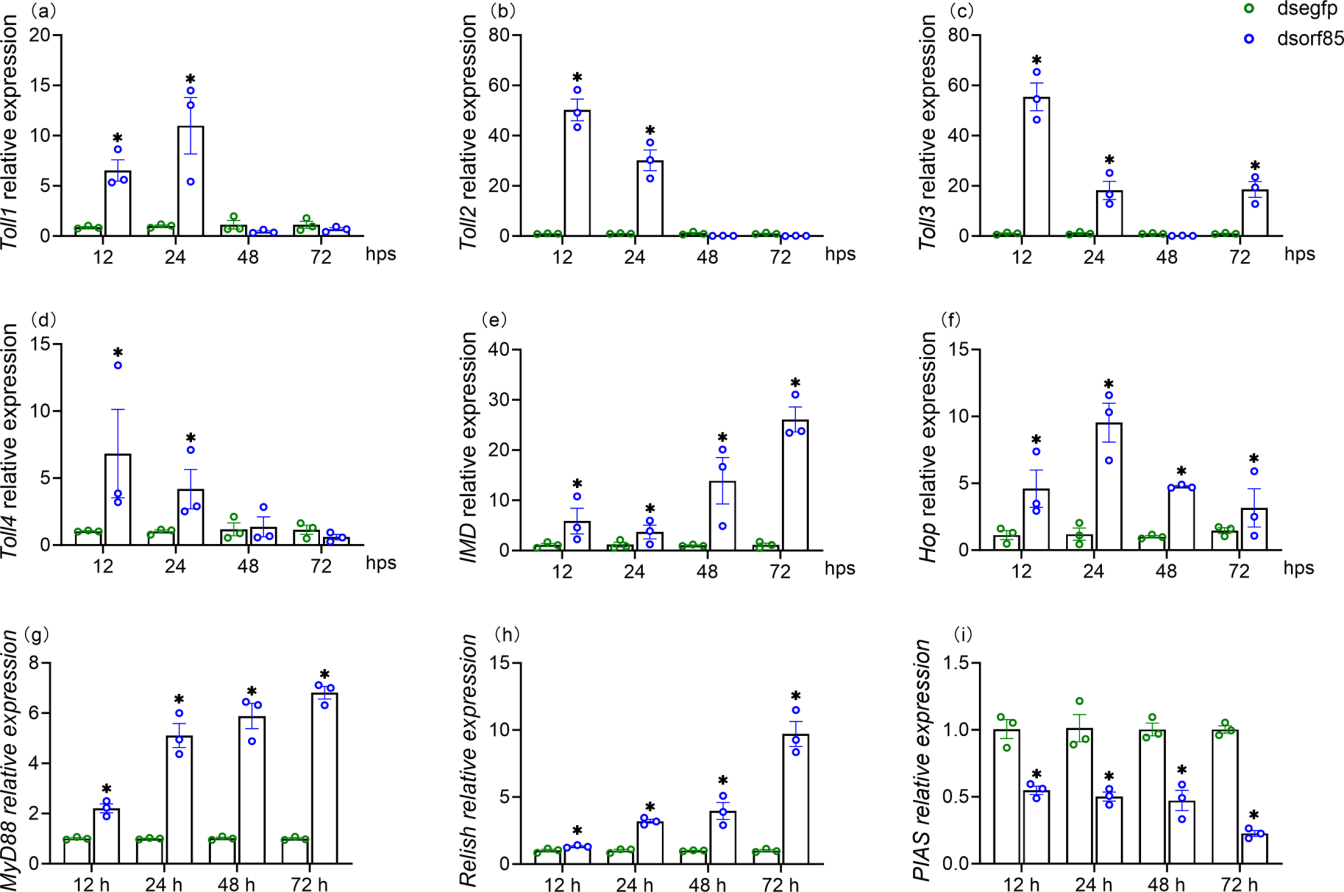
qRT-PCR quantification of some host innate immunity receptor and effector gene expression after silencing of the virus ORF85 gene expression. (**a–d**) Third-instar *S. exigua larvae* Toll gene relative expression level after HvAV-3h ORF85 gene expression silencing. (**e, f**) The host IMD and Hop gene’s relative expression, respectively. (**g–i**) Myd88, Relish and PIAS gene relative expression, respectively. Larvae injected with dsEGFP served as a control. Three biological replicates (each replicate consists of three individual larvae) were included at each tested time point. Error bars represent the standard error values. The unpaired Student *t*-test was used to analyse the significant difference between the obtained means. The star (*) symbol refers to the value of *P*<0.05.

Given that aegerolysin proteins are associated with diverse functions, including host-pathogen interactions, we were interested in examining if ORF85 gene silencing would affect the expression level of some host innate immunity-associated receptor or effector genes for different immunity pathways; for example, all four Toll receptors, Toll 1, 2, 3 and 4, were tested, along with IMD, Hop, MyD88, Relish and PIAS-encoding genes (Fig. 4). Interestingly, the qRT-PCR quantification revealed that the interference with this viral gene induced significant increases in some host immunity-associated genes in the three pathways. For example, all the tested Toll receptor genes were significantly induced by ORF85 silencing at the tested time points 12–24 hps (Fig. 4a–d). The range of induction was about 1.7- to 114-fold. Furthermore, the IMD receptor and Hop genes were upregulated after ORF85 gene silencing that ranged from about 1.8- to 44.7-fold and 2.1- to 22.9-fold, respectively, at the tested time points 12–72 hps (Fig. 4e, f). Additionally, we detected significant increases in the host MyD88 and Relish genes, alongside decreases in a PIAS gene (Fig. 4g–i). These findings reflect the importance of ORF85 in host-pathogen interaction and the possible association of this aegerolysin-like gene in host immune machinery suppression. However, the mechanism of this suppression needs further study.

### Cloning of the HvAV-3h ORF85 gene into the pET-28a vector and expression in *E. coli* cells

**Fig. 5.**
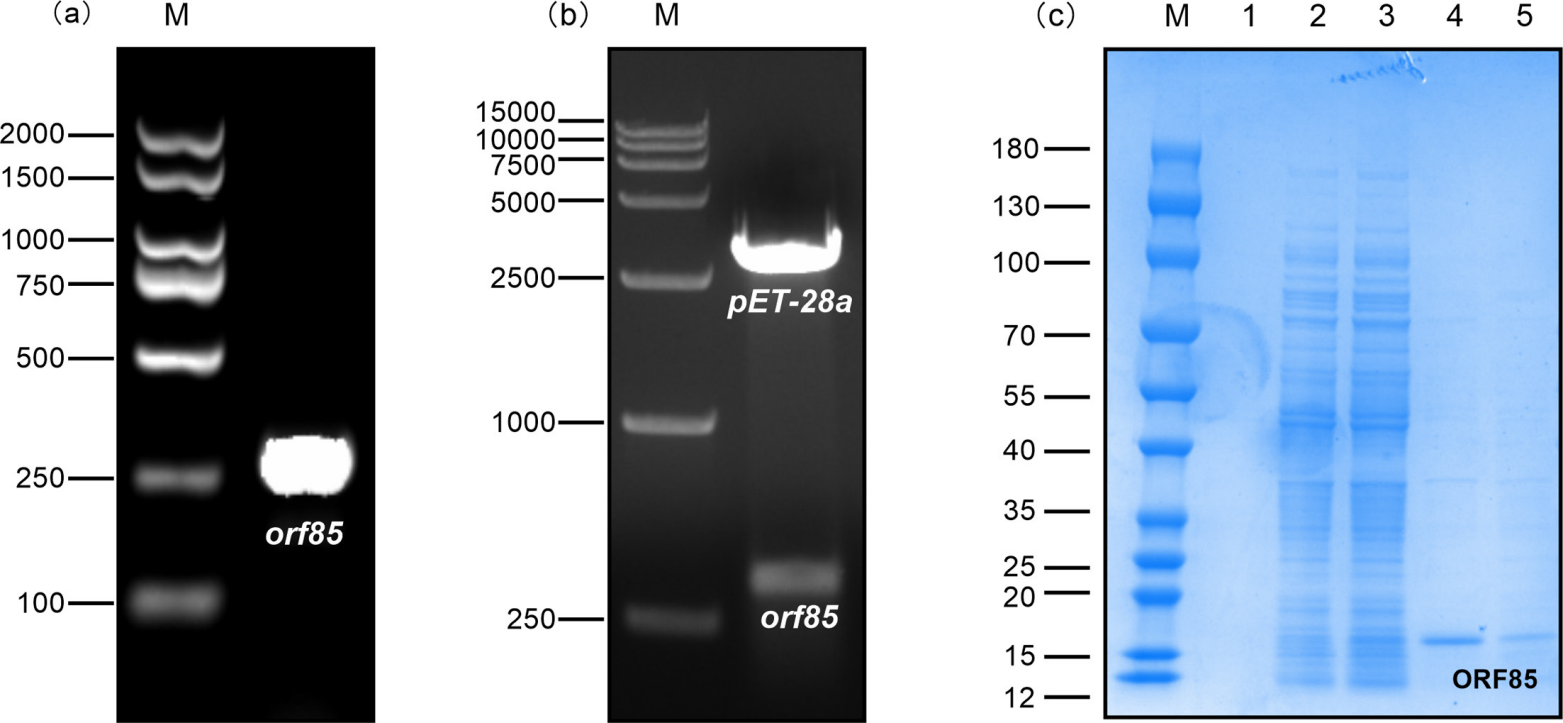
Expression and purification of the HvAV-3h ORF85 aegerolysin-like protein in *E. coli* cell BL21 (DE3). (**a**) PCR amplified ORF85 DNA fragment. M refers to the DNA marker/ladder (Vazyme, China). (**b**) BamHI and HindIII digestion of the constructed expression vector (pET-28a-ORF85) to confirm ORF85 gene insertion. (**c**) SDS-PAGE of expressed HvAV-3h His-*Tag protein and purified protein. Lane M refers to the 180 kDa Prestained Protein Marker (Vazyme, China). Lane 1 is loaded with the soluble cell lysate of *E. coli* cells transformed with pET-28a. Lane 2 is loaded with soluble cell lysate of pET-28a-ORF85-transformed *E. coli* cells before IPTG induction, whilst lane 3 is loaded with the soluble cell lysate transformed with the pET-28a-ORF85 after induction with 1 mM of IPTG for 18 h at 37 °C. Lanes 4 and 5 are loaded with the wash-out elution buffer of 10 mM and 50 mM imidazole, respectively.

The HvAV-3h ORF85 amplification by PCR using the cDNA synthesized from the total RNA collected from HvAV-3h-infected larvae led to the amplification of a 270 bp DNA fragment equivalent to the full length of this gene (Fig. 5a). The ORF85 PCR product and BamHI- and HindIII-digested pET-28a plasmid were gel-purified, eluted, ligated and transformed into *E. coli* BL21 DE3 for protein expression. The subsequent BamHI and HindIII digestion of the constructed plasmid pET-28a-ORF85 and the empty plasmid pET-28a confirmed the integration of the ORF85 gene (Fig. 5b). Furthermore, Sanger sequencing of the constructed plasmid reconfirmed the correct insertion of the aegerolysin-like gene into the expression vector. SDS-PAGE demonstrated the expression of a protein corresponding to the HvAV-3h ORF85-encoded protein and 6x-His*Tag (Fig. 5c). The highest expression level was achieved after 18 h of IPTG induction at 37 °C. A His-tag-fused EGFP protein was successfully expressed and purified following the same system and reagents (Fig. S1, available in the online Supplementary Material) to be used as a negative control. Western blotting confirmed the presence of His-tag-fused EGFP and HvAV-3h His-tag-fused aegerolysin protein using anti-His*Tag antibody (Figs S1 and S2). Moreover, a blood haemolysis test on 5% goat blood agar demonstrated the lack of lysis of blood cells and no clear-zone formation after 24 h of incubation at 25 °C (Fig. S3).

### The HvAV-3h aegerolysin-like protein possesses an antibacterial effect *in vitro* and reduces the host weight *in vivo*

**Fig. 6.**
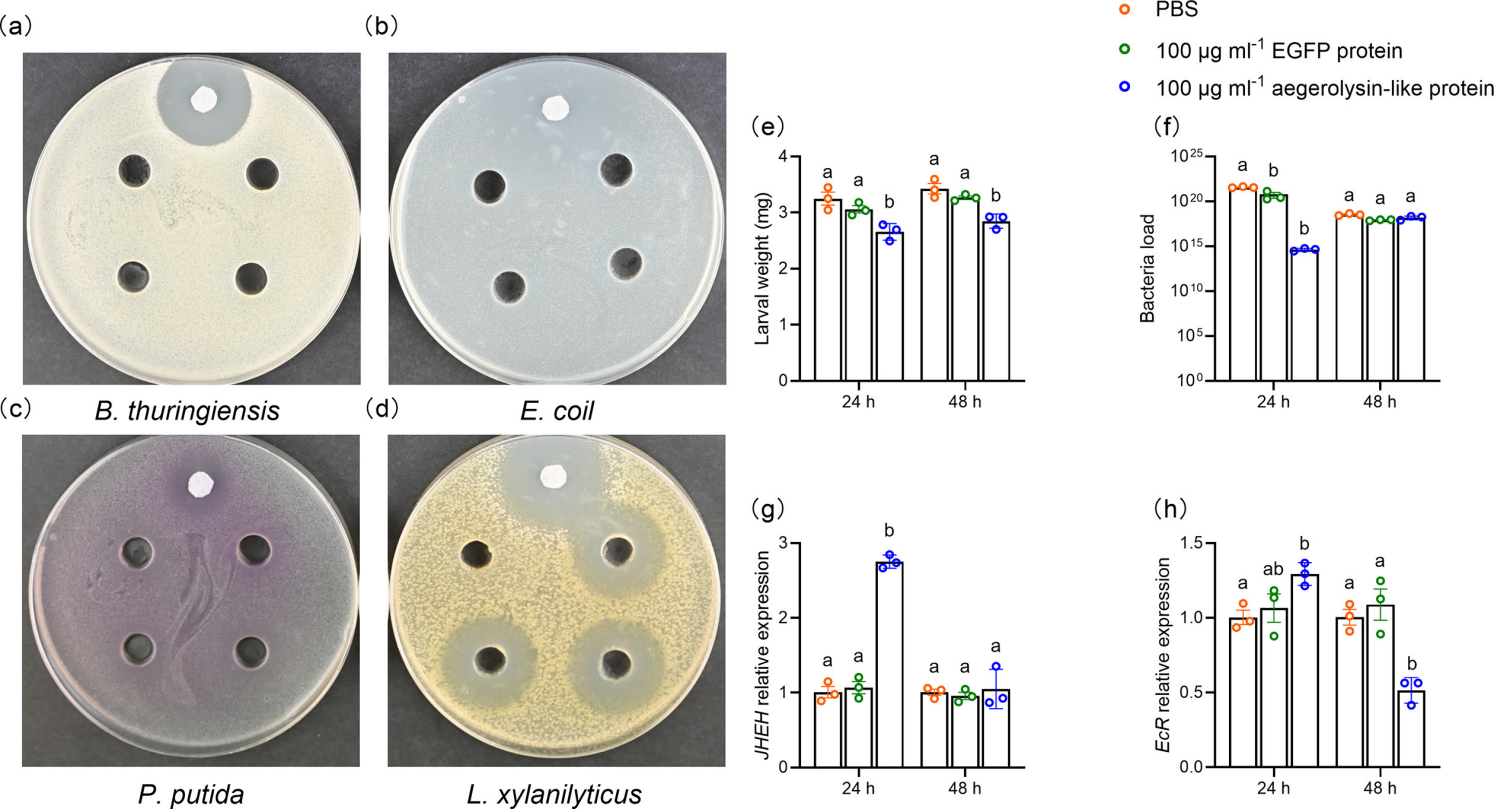
Determination of the HvAV-3h ORF85 recombinant protein antibacterial and growth retardation effects. (**a–d**) Antibacterial effect of the purified ORF85 recombinant protein (~340 µg ml^−1^) on *Bacillus thuringiensis*, *E. coli*, *Pseudomonas putida* and *L. xylanilyticus*. Wells with PBS buffer only served as a negative control (−). Filter paper saturated with chloramphenicol (25 mg ml^−1^) was used as a positive control (+). The antibacterial effect detected with *L. xylanilyticus* was further verified by examining an EGFP protein expressed by using the same methods and reagents (Fig. **S3**). (**e–h**) Growth index after feeding of purified ORF85 recombinant protein to the third-instar larvae *of S. exigua*. (**e**) Larval weight of third-instar *S. exigua* larvae after 24 or 48 h of the feeding of plant leaf discs immersed in HvAV-3h aegerolysin-like protein (100 µg ml^−1^), EGFP protein (100 ug ml^−1^) or PBS buffer. In (**e**), the dots on the histogram represent the mean of three biological replicates (15 larvae per replicate)*.* (**f**) Quantification of *S. exigua* gut bacterial load by qRT-PCR after feeding. (**g**) The relative quantification of *S. exigua JHEH* gene and (**h**) ecdysterone hormone gene (*EcR*) expression levels in HvAV-3h aegerolysin-like protein, EGFP protein or PBS-fed *S. exigua* larvae. In (f–h), the dots on the histogram represent the mean of three biological replicates (three larvae per replicate). Error bars represent the standard error values. The one-way ANOVA followed by the Tukey test was used to compare the obtained means of larval weight, bacterial load and hormonal gene expression level. The test was conducted using the SPSS 22.0 statistical software. Different letters refer to statistically significant differences between the obtained means.

We tested the antibacterial activity of the purified protein against four Gram-positive and Gram-negative bacterial species. The well-cut diffusion method revealed that the purified protein possessed a bactericidal effect only against *Lysinibacillus xylanilyticus* (Fig. 6a–d). The side effects of any residual purification reagents in the tested aegerolysin protein were verified by using an EGFP protein control purified using the same procedure and reagents (Fig. S3). Overall, the antibacterial effect of the HvAV-3h aegerolysin-like protein was confirmed under *in vitro* conditions.

Examining the purified viral aegerolysin-like protein feeding toxicity to third-instar *S. exigua* larvae revealed that this viral protein is not toxic by feeding, at least at the tested concentration (100 µg ml^−1^) to this insect. The mortality percentage was 0% after 24 or 48 h of HvAV-3h aegerolysin feeding. However, we observed a retardation in insect growth and development. For example, the mean insect’s weight gain was 2.65 and 2.84 mg after 24 and 48 h of the feeding of aegerolysin concentration (100 µg ml^−1^), respectively, in comparison to the EGFP-fed and PBS-fed control larvae that had 3.05–3.26 and 3.25–3.42 mg weight gain in the same time points, respectively (Fig. 6e). Moreover, the bacterial load in the gut of aegerolysin-fed larvae was reduced after 24 and 48 h post-feeding; however, the decrease was statistically insignificant (Fig. 6f). Oppositely, the relative expression of the *S. exigua JHEH* gene and the ecdysterone hormone gene (*EcR*) was increasing at 24 h post-feeding. However, the *EcR* demonstrated a significant decrease at 48 h post-feeding (Fig. 6g, h).

## Discussion

In the current study, we explored the biological role of a viral aegerolysin-like protein (ORF85) encoded by the insecticidal ascovirus HvAV-3h. Aegerolysins are found in a range of prokaryotes and eukaryotes; however, homologues of aegerolysin proteins are present uniquely in certain ascoviruses. Consistent with the multifunctional characteristics of aegerolysins identified in bacteria and fungi, we observed the HvAV-3h aegerolysin multifunctionality during the infection cycle of HvAV-3h in lepidopteran insect hosts.

The analysis of the HvAV-3h ORF85 protein sequence indicated that this viral protein structurally resembles various aegerolysin proteins found in fungi, bacteria, plants and animals. Amongst these proteins are those identified in *P. ostreatus*, *A. faecalis*, * P. aeruginosa*, *S. helianthus* and *P. patens*. Interestingly, experimental findings have demonstrated that these proteins are associated with diverse functions such as fungal fruiting [34], interaction with lipid membranes [35], insect toxicity [36] and the formation of membrane pores [37,38]. On the other hand, our phylogenetic study indicated that the HvAV-3h ORF85 protein is evolutionarily more closely related to aegerolysins found in lepidopteran insects. According to a previous evolutionary study, fungi are proposed to be the primary origin of aegerolysins [1]. It is hypothesized that horizontal gene transfer may account for the subsequent acquisition of aegerolysin-like proteins by certain bacteria and plants. The origin of the aegerolysin-like proteins encoded in the genomes of ascoviruses remains uncertain, as it is not clear whether the acquisition of genes for aegerolysin-like proteins was from the insect host or if the reverse is true [39].

Certain ascovirus species, such as HvAV and TnAV, encode the aegerolysin in their genomes, whereas other species, like SfAV, lack a homologous protein. To explore the effects of gene silencing on virus replication, we silenced this gene during an *in vivo* infection of two lepidopteran insects, *S. exigua* and *H. armigera*. Notably, the silencing of ORF85 was associated with reducing virus replication in the larvae of *S. exigua*. Moreover, histopathological examinations of HvAV-3h-infected third-instar *H. armigera* larvae, following injection of ORF85-specific dsRNA for different durations, demonstrated that the fat body tissue was less destroyed than unsilenced virus levels. These findings align with earlier Transmission Electron Microscope (TEM) examinations, which indicate that ascovirus species encoding aegerolysin exhibit a wide-tissue tropism, in contrast to species that do not encode aegerolysin, which are characterized by a limited tissue tropism [40,42]. On the other hand, HvAV-3h can infect a variety of lepidopteran insects, including *Spodoptera frugiperda*, *S. exigua*, *S. litura*, *H. armigera* and *Mythimna separata* [43]. While SfAV primarily targets members of the *Spodoptera* species [11]. Further research is necessary to determine whether aegerolysin facilitates cell entry or invasion. Nevertheless, based on our gene-silencing and histopathological examinations after interfering with HvAV-3h ORF85 gene expression, aegerolysin supports HvAV-3h replication and destruction of more fat body cells in infected lepidopteran larvae.

On the other hand, we have detected an increase of some innate immunity-associated receptor and effector genes after silencing the HvAV-3h ORF85, implying the role of this viral gene in regulating host immunity. For example, silencing of the aegerolysin gene was associated with significant increases for four *S. exigua* Toll receptor genes, an IMD receptor gene and a Hop gene. Additionally, we detected, in parallel, significant increases in the MyD88 and Relish genes. On the other hand, we detected a decline in the protein inhibitor of the activated STAT (PIAS) gene. Previous studies have identified MyD88 to function downstream of Toll-like receptors and the transcription factor Relish as an important factor required for activating the IMD pathway target genes [44,45]. Moreover, a decrease in PIAS level was associated with increased immunity against bacterial and viral infections [46]. All these observations combined confirm the association of this viral-encoded protein in regulating the host immune system. However, further studies will be needed to determine whether the changes in innate immunity gene expression are a direct result of silencing this viral gene or if they represent indirect consequences of its antibacterial properties, which are anticipated to influence the host microbiome and immune system. Previous transcriptomic studies have generally indicated that ascovirus infection alerts different immune pathways; however, this immune response was insufficient to clear the infection. Consequently, infected larvae may survive for several weeks following infection, suggesting that the virus possesses some genes to suppress this immune response, like the diedel gene identified in the SfAV-1a transcriptome study [9,14, 15].

Due to the entomopathogenic nature of ascoviruses and the established insecticidal properties of aegerolysin proteins derived from other sources against certain insects [19], we aimed to investigate the insecticidal toxicity of the purified recombinant HvAV-3h aegerolysin protein on *S. exigua* larvae. Whilst we did not detect any insecticidal toxicity from HvAV-3h aegerolysin when fed to third-instar *S. exigua* larvae, the larvae that were fed suffered from other growth retardation symptoms. The symptoms included a significant reduction in larval weight and an imbalance in *JHEH* and ecdysterone hormone gene expression levels. On the other hand, we did not detect a statistically significant decline in bacterial load within the gut of larvae fed with aegerolysin; however, we detected an antibacterial effect for the HvAV-3h recombinant protein against the *L. xylanilyticus* through the well-cut diffusion method. This bacterial species has been identified in a recent study as a gut-resident bacterium in *Protaetia brevitarsis seulensis* larvae [47]. Previous studies conducted on other aegerolysin-like proteins derived from other prokaryotic or eukaryotic sources revealed that these proteins possess specific antimicrobial actions. For instance, eryngeolysin exhibited antibacterial activity against *Bacillus subtilis* and *Bacillus megaterium*. On the contrary, it was ineffective against *Staphylococcus aureus*, *E. coli*, * P. aeruginosa*, *Pseudomonas fluorescens*, *Proteus vulgaris*, *Mycobacterium phlei* and *Enterobacter aerogenes*. Moreover, eryngeolysin did not demonstrate antifungal activity against different fungal species [48]. In the case of ascoviruses, it is also important to consider the route of transmission in nature. Previous studies demonstrated that ascoviruses are mainly transmitted mechanically by female wasps, with lesser transmission occurring by feeding [11]. Therefore, aegerolysins are likely to primarily affect bacteria associated with the host haemolymph or fat body tissues rather than those associated with the gut during virus replication.

The role of some viral proteins as antimicrobials or cell-penetrating peptides is gaining interest, particularly as an increasing number of these proteins are being discovered in various viruses through both computational and experimental methods. Moreover, they are biotechnologically novel and underexplored natural resources for developing new AMP and CPPs. For example, 426 and 2,444 antimicrobials and cell-penetrating peptides, respectively, were identified *in silico* after analysing 133 viruses [49]. Viral CPP examples were detected in HIV-1 [50], herpes simplex and herpesvirus 8 [24,51], hepatitis B [52], Flock House virus [53] and dengue virus [54]. Generally, cell-penetrating peptides are more common than AMP viral proteins [49,55, 56]. However, AMPs were reported for some viral proteins encoded by the hepatitis B virus, Bean golden mosaic virus and some bacteriophages such as Bacillus phage SPbeta and Streptococcus phage Cp-1 [57].

In conclusion, our study on the HvAV-3h aegerolysin-like protein indicates that the HvAV-3h ORF85 plays a multifunctional role during the ascovirus infection in its insect host. The aegerolysin provides HvAV-3h with certain species-specific traits, thereby enhancing the virus’s ability to replicate and damage more host cells whilst suppressing the host immune response. Furthermore, experiments involving insect feeding and the treatment of some Gram-positive and Gram-negative bacterial species with HvAV-3h aegerolysin have demonstrated the antimicrobial properties of this protein. Overall, antimicrobial proteins derived from insecticidal viruses constitute a biotechnologically innovative and relatively underexplored resource within this class of proteins.

## Supplementary material

10.1099/jgv.0.002107 Uncited Supplementary Material 1.

## Abbreviations

CPP: cell-penetrating protein
EcR: ecdysone receptor
hps: h post-silencing
HvAV-3h: Heliothis virescens ascovirus 3h
IPTG: isopropyl-β-d-thiogalactoside
JHEH: juvenile hormone epoxide hydrolase
LB: Luria–Bertani
NJ: neighbour-joining
PDB: Protein Data Bank
qRT-PCR: quantitative reverse transcription PCR
SfAV-1a: Spodoptera frugiperda ascovirus 1a
StnII: sticholysin II
TnAV-6a1: Trichoplusia ni ascovirus
