# Supplementary material for: An aegerolysin-like protein from Heliothis virescens ascovirus 3h (HvAV-3h) shows immune suppression and antibacterial activity
Source: J Gen Virol. 2025 May 29;106(5):002107. doi: 10.1099/jgv.0.002107 (PMC12163727; doi:10.1099/jgv.0.002107)
Supplement: Uncited Supplementary Material 1. [file jgv-106-02107-s001.pdf]

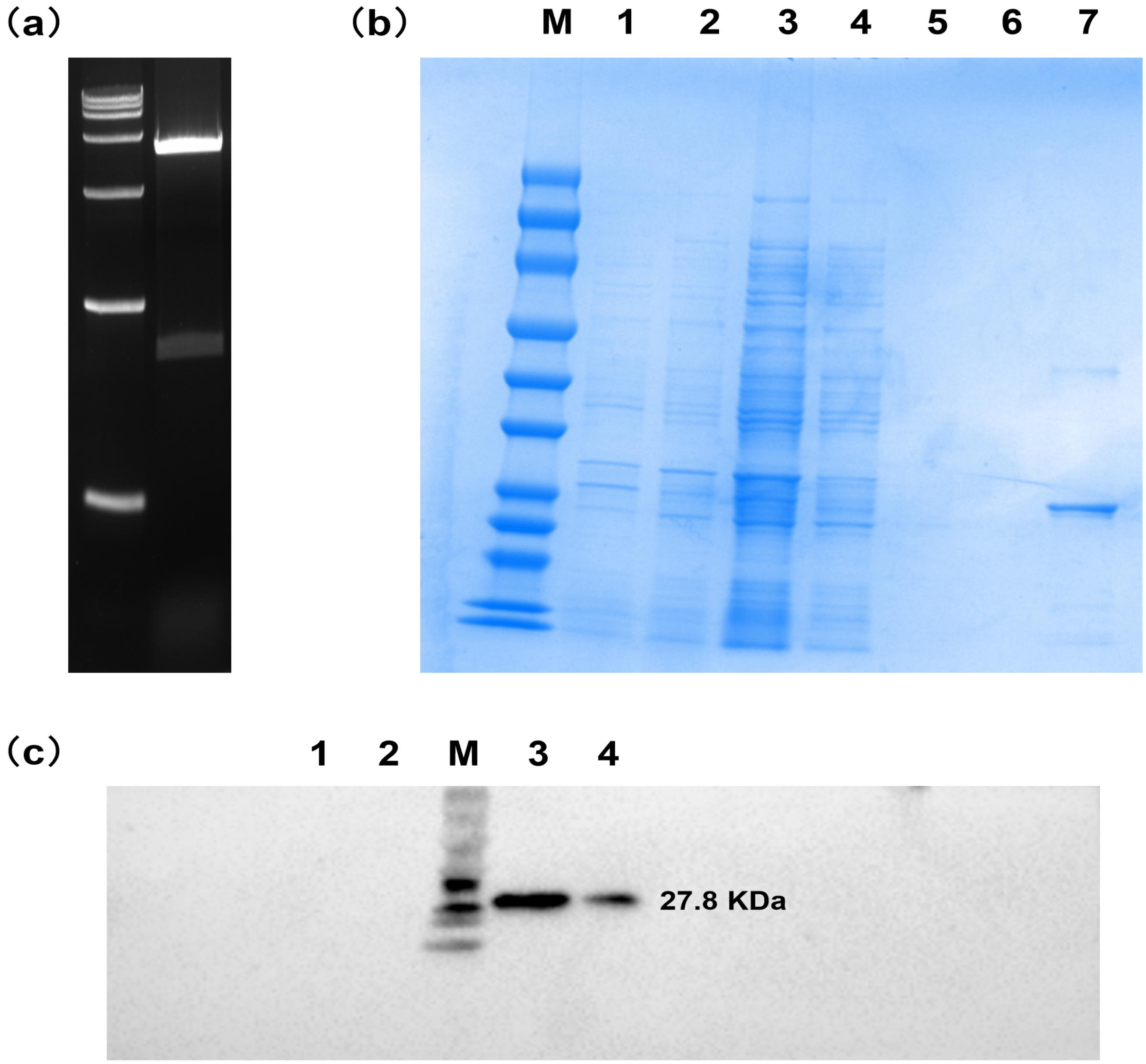

Fig. S1: Expression and purification of the Enhanced Green Fluorescent Protein (EGFP) in *E. coli* cells BL21(DE3). (a) The NotI and XhoI digestion of the constructed expression vector (pET-28a-egfp) to confirm *egfp* synthetic gene insertion. M refers to the DNA Marker/Ladder (Vazyme, China). (b) SDS-PAGE of expressed and purified His-tag fused EGFP. Lane M refers to the 180 kDa Prestained Protein Marker (Vazyme, China). Lane 1 is loaded with the soluble cell lysate of *E. coli* cells transformed with pET-28a plasmid vector. Lane 2 is loaded with the soluble cell lysate of pET-28a-EGFP transformed *E. coli* cells before isopropyl-β-d-thiogalactoside (IPTG) induction. Lanes 3 and 4 are loaded with the soluble cell lysate of pET-28a-EGFP transformed *E. coli* cells induced with one millimolar IPTG for 18 h at 37 °C. Lanes 5, 6, and 7 are loaded with the wash-out elution buffer of 10 mM, 50 mM, and 100 mM imidazole, respectively. (c) Western blot analysis of the purified His-tag fused EGFP protein using anti-His tag antibodies. M refers to the 180 kDa Prestained Protein Marker (Vazyme, China). Lanes 1 and 2 refer to the western blot assay on pET-28a transformed *E. coli* cells, and Lanes 3 and 4 refer to the western blot assay on purified His-tag fused EGFP protein.

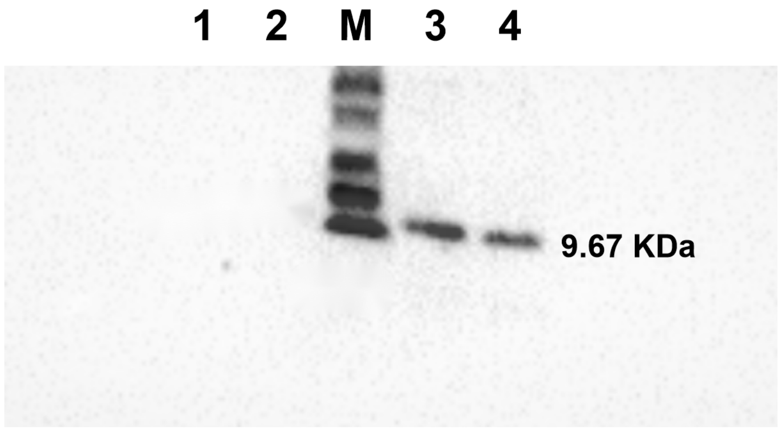

Fig. S2: Western blot of the purified Heliothis virescens ascovirus 3h (HvAV-3h) His-tag fused aegerolysin-like protein (ORF85-encoded protein) using anti-His antibody. M refers to the 180 kDa Prestained Protein Marker (Vazyme, China). Lanes 1 and 2 refer to the western blot assay on pET-28a transformed *E. coli* BL21 (DE3) cells, and Lanes 3 and 4 refer to the western blot assay on purified His-tag fused aegerolysin protein.

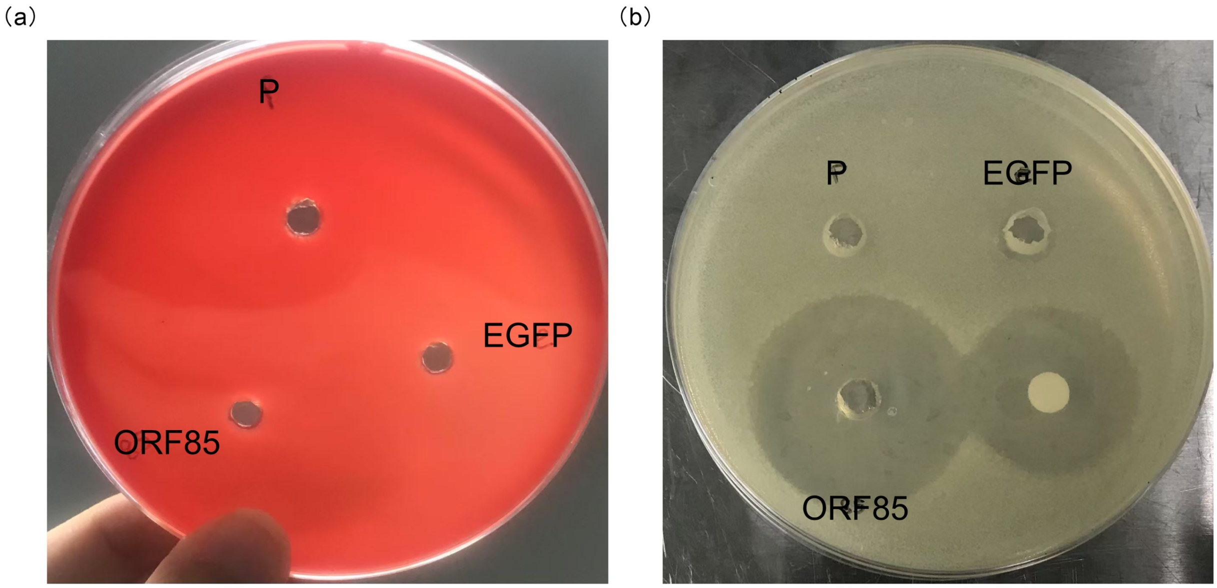

Fig. S3: Examination of Heliothis virescens ascovirus 3h (HvAV-3h) purified aegerolysin protein (100 mg/ml) effect on blood cells hemolysis and *Lysinibacillus xylanilyticus* bacterial growth. (a) hemolytic activity of the purified aegerolysin protein was examined on the surface of a 5% goat blood agar plate. The purified protein did not cause blood hemolysis. (b) antibacterial effect of the purified aegerolysin protein on *L. xylanilyticus.* An EGFP protein expressed and purified using the same protocol and reagents and a phosphate-buffered saline (PBS) buffer only served as negative controls for both tests. P letter refers to the PBS-containing well. The antibiotic disc in b refers to Ampicillin (AMP, 10) positive control. In b, notice the slight inhibitory effect around the EGFP-containing well (100 mg/ml) caused by possible protein purification residual chemicals. The test was made in triplicates in each case.
